# An instrumented bone/soft tissue phantom designed to mimic HIFU treatments of bone

**DOI:** 10.1186/2050-5736-3-S1-P1

**Published:** 2015-06-30

**Authors:** Jemma Brown, Gail ter Haar

**Affiliations:** 1 Institute of Cancer Research, London, United Kingdom

## Background/introduction

Clinically, HIFU exposures of bone are used to palliate pain from primary or secondary bone tumours. Such tumours weaken bone structure and render the patient susceptible to bone fracture. Ultrasound metrology for bone exposures is extremely challenging. The ultrasound beam is strongly reflected at the bone surface, with rapid surface absorption of sound entering the cortex. Pain relief is obtained when the HIFU induced temperature increase ablates the peri-osteal nerves. Standard PRF based MR thermometry, used for treatment monitoring, is inappropriate for bone. Thus, a method of determining the temperature distribution in a clinical environment is needed.

## Methods

An instrumented bone phantom which will allow HIFU exposure levels to be quantified, is being developed to address the problems described above. This phantom will initially be characterised in terms of its thermal, acoustic, and MR properties. Probes which allow simultaneous temperature and pressure measurements are being incorporated into the phantom. These will allow real-time mapping of these parameters under clinically realistic conditions. Access to the “research platform” on the ICR’s Sonalleve system will allow control of FUS exposure conditions, in this clinically relevant setting. The phantom composition is determined by subjecting fresh bone, and potential bone mimicking samples (with adjacent *ex vivo* soft tissue or soft tissue mimics) to varying levels of ultrasound exposures. Measurements of acoustic pressure and temperature at bone surfaces are compared to allow identification of the best bone mimic. The differences between wet and dry bone, in acoustic and thermal terms, are to be determined in order to gauge the viability of using animal bone as the mimic material. Soft tissues alone will be subjected to similar exposures in an effort to understand the effect of bone on the acoustic field in terms of delivered treatment. Simulated acoustic field and temperature distributions will be compared with those determined experimentally. The most appropriate phantom geometry (in terms of the dimensions of bone and overlying tissue paths) is being determined from patient CT scans. If deemed necessary, different geometries, representative of different anatomical sites will be used. Compromises must be made in order to determine the temperature distribution throughout the bone phantom accurately, whilst not changing its structural integrity significantly. Techniques such as infrared imaging and the use of thermochromatic crystals are being employed to provide surface temperature measurements. Optical fibre probes are being used for more invasive measurements. These are safe in an MR environment, avoid the problems associated with metallic thermocouples and can be used to simultaneously measure temperature and acoustic pressure. The effects of ultrasound exposure of simple fresh *ex vivo* tissues or 3D cell containing gel matrices, will be compared with samples that contain bone and/or bone phantom, using simulation, pathological and thermometric studies.

## Results and conclusions

An instrumented bone phantom will be developed. This will provide a valuable tool for assisting in planning of bone treatments, and it is hoped that this will help to accelerate their increased clinical availability.

